# Next-Generation Dengue Vaccines: Novel Strategies Currently Under Development

**DOI:** 10.3390/v3101800

**Published:** 2011-09-26

**Authors:** Anna P. Durbin, Stephen S. Whitehead

**Affiliations:** 1 Center for Immunization Research, Department of International Health, Johns Hopkins Bloomberg School of Public Health, 624 N. Broadway, Room 251, Baltimore, MD 21205, USA; 2 Laboratory of Infectious Diseases, National Institute of Allergy and Infectious Diseases, National Institutes of Health, 33 North Drive, Room 3W10A, Bethesda, MD 20892, USA; E-Mail: swhitehead@niaid.nih.gov

**Keywords:** Dengue vaccine, DNA vaccine, vectored-vaccine, sub-unit protein vaccine

## Abstract

Dengue has become the most important arboviral infection worldwide with more than 30 million cases of dengue fever estimated to occur each year. The need for a dengue vaccine is great and several live attenuated dengue candidate vaccines are proceeding through clinical evaluation. The need to induce a balanced immune response against all four DENV serotypes with a single vaccine has been a challenge for dengue vaccine developers. A live attenuated DENV chimeric vaccine produced by Sanofi Pasteur has recently entered Phase III evaluation in numerous dengue-endemic regions of the world. Viral interference between serotypes contained in live vaccines has required up to three doses of the vaccine be given over a 12-month period of time. For this reason, novel DENV candidate vaccines are being developed with the goal of achieving a protective immune response with an immunization schedule that can be given over the course of a few months. These next-generation candidates include DNA vaccines, recombinant adenovirus vectored vaccines, alphavirus replicons, and sub-unit protein vaccines. Several of these novel candidates will be discussed.

## 1. Introduction

Dengue viruses belong to the *Flaviviridae* family and present as four distinct serotypes; DENV-1, DENV-2, DENV-3, and DENV-4, with each serotype capable of causing the full spectrum of dengue illness [1]. It is currently estimated that there are more than 3.6 billion people at risk for dengue infection with 36 million cases of dengue fever, more than 2 million cases of severe dengue, and more than 21,000 deaths occurring each year [2]. Epidemiological studies have determined that the risk for more severe dengue illness is higher following a second, heterotypic DENV infection than for a primary DENV infection [3,4], although severe illness can occur following primary infection. Potential immune enhancement resulting from prior infection is thought to increase virus replication, which has been shown to correlate with disease severity [5]. Although severe dengue illness can occur with a third or fourth DENV infection, this risk appears to be very low [4]. For these reasons, there is consensus that a successful DENV vaccine must ideally protect against all four DENV serotypes. Additional characteristics of the ideal dengue vaccine are the ability to induce long-lived immunity to all four DENV serotypes with a limited number of doses given over a period of weeks to a few months and affordability such that the vaccine can be made available to the populations most at risk for dengue infection.

Infection with one DENV serotype is thought to induce long-lived if not life-long homotypic immunity but only short-term heterotypic immunity [6]. Neutralizing antibody against the envelope (E) glycoprotein is the main determinant of protection against dengue and therefore, induction of neutralizing antibody against all four DENV serotypes is the target for dengue vaccines [7]. Antibody responses against prM and NS1 have also been identified as protective [8,9]. The E protein is comprised of three domains, I, II, and III. It is the epitopes on the surface of domain III that primarily elicit serotype-specific neutralizing antibody responses and for this reason, antigens comprised of domain III epitopes have been proposed as candidate vaccines [10]. In order to prevent irreversible conformational change of E as it is processed through the acidic trans-Golgi network, E must be expressed with prM and thus, most sub-unit dengue vaccines have utilized prM and E as the antigens of choice [11].

The DENV vaccines furthest along in clinical development are live attenuated vaccines (LAV). Several investigational tetravalent LAV candidates are currently being evaluated in clinical trials [12–14]. However, developing a tetravalent LAV that is sufficiently attenuated for each of the monovalent components yet immunogenic for all four dengue serotypes has been a challenge [13,15–17]. Despite these challenges, Phase III efficacy trials of a live attenuated tetravalent chimeric dengue vaccine based on the yellow fever 17D virus (CYD) produced by Sanofi Pasteur are underway in a number of dengue-endemic areas. In addition, a live attenuated recombinant tetravalent dengue vaccine developed by the U.S. National Institutes of Health has been evaluated in a number of Phase I studies and will soon begin clinical evaluation in Brazil and a live attenuated chimeric vaccine produced by Inviragen has entered Phase I clinical trials. These candidates have been well described elsewhere and will not be discussed here [12,18–21].

A challenge to live attenuated dengue vaccine development is the ability to induce a balanced immune response to all four serotypes. It is thought that viral interference, whereby one vaccine virus serotype outcompetes the others resulting in the induction of neutralizing antibodies to that virus at the expense of the others, may be responsible for this unbalanced antibody response [15,22,23]. A strategy to overcome viral interference/immunodominance is to deliver multiple doses of the live candidate vaccine. For instance, three doses of the CYD vaccine given over a 12-month period of time to dengue-naïve children and adults induced neutralizing antibody to all four serotypes in more than 60% of vaccinees [18]. A second approach being developed to induce a balanced antibody response to all four serotypes over a compressed dosing schedule is to use non-propagating candidate vaccines that express the protective antigens of the four DENV. This article will focus on non-live attenuated dengue vaccine candidates that have shown promise in preclinical evaluation but have not started clinical evaluation or are in the very early stages of clinical evaluation.

Several novel dengue vaccine candidates have been developed including DNA vaccines, viral-vectored candidates, and sub-unit protein vaccines (Table 1). These candidates have several potential advantages over replicating LA dengue vaccine candidates. The DNA vaccines, non-propagating viral vectored candidates, and sub-unit protein vaccines discussed below do not replicate in mammalian cells, allowing for their administration to immunocompromised hosts, a general contraindication for live replicating vaccine viruses. Recombinant poxviruses and adenoviruses expressing foreign proteins have been demonstrated to induce strong humoral and cellular responses in humans against various pathogens [24–26]. DNA vaccines and viral-vectored dengue vaccines express their proteins *de novo* within the cell, and because of intracellular translation and processing of the gene products, MHC class I dependent immune responses can be induced in addition to humoral immunity.

## 2. Next Generation Vaccines

### 2.1. DNA Vaccines

DNA vaccines have the potential to induce humoral and cellular immune responses similar to those of live attenuated vaccines described above but without some of the safety issues inherent to live vaccines. They offer the additional advantage of very low cost and excellent stability over a wide-range of temperature and for long periods of time. The first dengue DNA vaccine developed utilized the plasmid p1012D2ME to express the prM signal sequence, prM, and 92% of the DEN2 NGC E protein truncated at the carboxy terminus to enhance secretion of E [27]. Mice were inoculated intramuscularly or intradermally on study days 0, 9, 22, and 57. In intradermally inoculated mice, neutralizing antibody was detected out to 11 months. This vaccine was later administered with a recombinant fusion protein containing 103 amino acids of the Domain III of the DEN2 envelope protein fused to the maltose-binding protein (MPB) of *E. coli* in a prime-boost protocol [29]. Three doses of the DNA vaccine given with the recombinant fusion protein (RD/RD/RD) were compared to three doses of the DNA vaccine alone (D/D/D), three doses of the recombinant fusion protein alone (R/R/R), the recombinant fusion protein followed by two doses of the DNA vaccine (R/D/D), or the DNA vaccine followed by two doses of the recombinant protein (D/R/R). The RD/RD/RD and D/R/R groups developed much higher neutralizing antibody titers than did the D/D/D or the R/D/D groups. Unfortunately, when this strategy was later evaluated in non-human primates, no protection against viremia was observed in animals that received the DNA vaccine with or without the recombinant fusion protein alone or as part of a prime-boost protocol [28].

The DENV-2 DNA vaccine was later administered with a plasmid expressing mouse CpG motifs, significantly enhancing the neutralizing antibody response of the DNA vaccine [30]. A further attempt to improve the neutralizing antibody response induced by the DENV-2 DNA vaccine was made by constructing a plasmid that expressed the DENV-2 prM and E protein fused to the carboxy-terminal sequence of the lysosome-associated membrane protein (LAMP) [31]. In this experiment, fusion of the prM-E to LAMP effectively targeted the protein to the lysosomal compartment of NIH3T3 cells. Targeting of protein to the lysosomal compartment for degradation would presumably allow for MHC class II presentation and therefore, an enhanced CD4^+^ T helper cell and antibody response. Mice immunized with the prM-E-LAMP DNA construct developed higher antibody titers than those immunized with the prM-E construct. This response was further augmented when the prM-E-LAMP construct was administered with a second plasmid that expressed mouse GM-CSF [31]. Not only were higher neutralizing antibody titers induced by the prM-E-LAMP DNA vaccine, but the antibody titers persisted for nearly 1 year post-vaccination [32].

A DENV-1 DNA vaccine expressing the prM and carboxy-truncated or full-length E was evaluated in mice [33]. Of all the constructs, the DNA vaccine expressing prM and full-length E induced the best neutralizing antibody response. This construct was then administered intradermally (ID) or intramuscularly (IM) to Aotus monkeys at time 0 and then boosted at 1 and 5 months, followed by challenge with wild-type DENV-1 at 11 months [35]. The DEN1 DNA vaccine administered by the ID route induced higher neutralizing antibody titers and greater protection against challenge than that administered by the IM route [35]. However, when this vaccine was administered to rhesus macaques with a pUC19 plasmid expressing CpG motifs, there was no significant difference in the neutralizing antibody titer or protection against wild-type DENV-1 challenge compared to the group that received the DNA vaccine without the pUC19 plasmid, possibly due to differences in non-human primate CpG motifs compared with mouse CpG motifs [34]. To test this hypothesis, the DENV-1 DNA vaccine was administered to Aotus monkeys alone, with a plasmid (pHis64) containing multiple copies of human immunostimulatory sequence (ISS) motifs, or with pHis64 and a plasmid expressing Aotus GM-CSF [36]. Monkeys were given 3 doses of vaccine (0, 1, and 5 months) and then challenged with wild-type DENV-1 at 6 or 11 months. The formulation containing both the GM-CSF and ISS-expressing plasmids in addition to the DNA vaccine did not increase neutralizing antibody titers significantly but did afford 100% protection to animals challenged 6 months after vaccination. In a proof-of-concept Phase I clinical trial of this DNA vaccine alone, 12 healthy adult subjects received three vaccinations of 1.0 mg of plasmid DNA expressing the prM and E genes of DENV-1 and 12 subjects received 5.0 mg of plasmid DNA expressing the prM and E genes of DENV-1. The dosing interval was 0, 1, and 5 months and the vaccine was administered using a needle-free device [37]. Unfortunately, the DNA vaccine was poorly immunogenic in humans with only 5/12 subjects in the high-dose group producing measurable neutralizing antibody. This vaccine may be further evaluated as part of a prime-boost strategy.

Konishi *et al.* developed a tetravalent DNA vaccine by combining four plasmids, each expressing the signal-prM-E sequence of one of the four DENV serotypes [38,39]. A mixture comprised of 25 mg of each plasmid induced a fairly well-balanced neutralizing antibody response to all four DENV serotypes in mice; the response was heightened when the interval between first and second immunization was increased from 3 to 7 weeks [39]. The antibody response to the four DENV serotypes induced by the DNA vaccines was further boosted 2- to 4-fold when an inactivated JE vaccine or subunit protein DEN2 vaccine was administered with the DNA vaccine [40]. Studies to evaluate the protective efficacy of this tetravalent dengue DNA vaccine co-administered with JE-Vax or a sub-unit DENV-2 vaccine are currently being planned in non-human primates.

The technique of DNA shuffling was utilized to develop DNA chimeric vaccine constructs encoding antigens comprised of prM and E epitopes of all four DENV serotypes in a single construct [41,42]. Seven out of the 67 clones that were chosen for screening induced neutralizing antibody to all four DENV serotypes in mice; three of these clones protected 80%–100% of mice from lethal DENV-2 challenge [41]. Macaques were vaccinated with 5 mg of one of the three shuffled DNA clones or a mixture of all three shuffled DNA clones on study day 0, 28, and 84. Four weeks after the third dose, variable neutralizing antibody responses were detected in animals vaccinated with the individual shuffled DNA clones with only one clone inducing neutralizing antibody to all four DENV serotypes [42]. Higher neutralizing antibody titers were induced to all four DENV serotypes by the formulation containing all three shuffled DNA clones. Animals were challenged at week 32 with wild-type DENV-1 or DENV-2. Disappointingly, only partial protection was observed against DENV-1 and no protection against DENV-2 was observed. These constructs may be further developed by the incorporation of immunostimulatory sequences or adjuvants to improve the immune response.

### 2.2. Adenovirus-Vectored Vaccines

Although DNA vaccines have shown promise in mouse models, the potency of these vaccines has been somewhat disappointing, as discussed above. The limited success of DNA vaccines is thought to be due to their poor uptake and limited expression in the host cell. To improve the delivery and expression of antigenic sequence, several viral vector platforms have been evaluated as candidate dengue vaccines, including adenovirus vectors, attenuated vaccinia vectors, and alphavirus vectors. Recombinant replication-deficient adenovirus (Ad) vectors can express large quantities of antigen, making them extremely immunogenic. Because of their ability to induce potent humoral and cellular immune responses, they have been utilized to develop candidate vaccines for a variety of pathogens, including dengue [43–45,55–58]. Complex adenovirus vectors (cAd) have been developed by GenPhar Inc. that contain large deletions in the adenoviral E1, E3, and E4 regions, allowing for the incorporation of large amounts of foreign DNA. Therefore, these cAd vectors are capable of accommodating the prM and E glycoprotein complex of two DENV in a single vector [43,44]. cAdVaxD(1-2) contains the prM-E of DENV-1 and DENV-2 at opposite ends of the vector and cAdVaxD(3-4) contains those of DENV-3 and DENV-4. Both cAdVaxD vectors were found to be immunogenic in mice and were then evaluated in a non-human primate model. Rhesus macaques received doses (10^9^ infectious units each) approximately 2 months apart of a tetravalent vaccine formulated by mixing the two vectors cAdVaxD(1-2) and cAdVaxD(3-4) [45]. Animals were challenged with wild-type DENV at either 4 or 24 weeks following vaccination. Although only 22% of animals had a tetravalent neutralizing antibody response following the first dose of vaccine, all animals developed a tetravalent neutralizing antibody response following the second dose. Those animals challenged at 4 weeks were completely protected against DENV-1 and DENV-3 and demonstrated minimal breakthrough viremia for DENV-2 (1 animal for 1 day) and DENV-4 (3 animals for 1 day). Animals challenged 24 weeks after vaccination were also completely protected from DENV-1 and DENV-3, and significant protection against DENV-2 and DENV-4 was also induced. However, 3/4 animals challenged with DENV-4 were viremic and the titer of viremia appeared higher than that of control animals. Although animals developed anti-adenovirus antibodies following the first dose of vaccine, the second dose was able to boost both dengue and adenovirus antibodies suggesting that the antibody did not inhibit the second dose. Based on these results, the authors believe further evaluation of these constructs in a clinical trial is warranted.

A heterologous prime-boost strategy involving a bivalent rAd vector (rAd-C) encoding a chimeric antigen comprised of the envelope domain III of DENV-2 and DENV-4 was evaluated in mice [46]. Mice were immunized with 10^8^ PFU of rAd-C and then boosted 15 days later with a plasmid expressing the same chimeric antigen (pVAX-EDIII-4/2). A balanced neutralizing antibody response as well as a T cell response specific to DENV-2 and DENV-4 was induced in the mice suggesting that this strategy could overcome viral interference. Work is ongoing to construct a similar bivalent rAd vector for DENV-3 and DENV-1 with the hope of eventually developing a tetravalent DENV vaccine.

### 2.3. Alphavirus Replicon Vaccines

Non-propagating Venezuelan equine encephalitis virus replicon particles (VRP) are being evaluated by the Carolina Vaccine Institute as a platform for DENV vaccine development. VRPs have several attributes including their ability to express high levels of a foreign gene in a single round of infection, their inherent adjuvant activity, and their efficient infection of dendritic cells [59,60]. A single 10^6^ IU dose of VRP expressing the prM-E proteins of DENV-2 NGC induced neutralizing antibody in 3-week-old mice, even in the presence of maternal anti-DENV antibody [47]. To assess the ability of the VRP to protect against lethal DENV challenge, BALB/c mice received a single dose of the DENV-2 VRP at 3 weeks of age or two immunizations 2 weeks apart (at age 3 weeks and 5 weeks). Doses ranging from 10^4^–10^6^ IU were evaluated. The 10^6^ dose of the DENV-2 VRP completely protected all mice from DENV2 challenge, whether they had received one or two doses. Lower doses induced high level of protection but did not completely protect all mice. Interestingly, 11/12 pups born to DENV-2 VRP-vaccinated dams were also protected from a lethal DENV-2 challenge administered three weeks after birth. A second VRP expressing the soluble E protein of DENV-3 was evaluated in rhesus macaques [48]. Animals received three immunizations containing 10^8^ IU of the DENV-3 VRP at week 0, 7, and 18 and were then challenged 15 weeks after the third immunization. Neutralizing antibody to DENV-3 was induced following the first immunization and all animals were completed protected from wild-type DENV-3 challenge. Evaluation of a tetravalent DENV-VRP in non-human primates is planned.

### 2.4. Recombinant 80E Sub-Unit Protein Vaccine

The *Drosophila S2* system is able induce high level expression of proteins of interest and was therefore chosen to express a plasmid containing the prM and N-terminal 80% of the E gene sequence of DENV-2 [49]. The produced polyprotein is then cleaved by endogenous proteases to release the 80E protein with a native-like N terminus. Two doses of the DENV-2 sub-unit 80E protein were administered to rhesus macaques in combination with one of seven different adjuvants at a three-month dosing interval [49]. Animals were challenged with wild-type DENV-2 two months after the last dose of vaccine. All animals developed detectable neutralizing antibody after the first dose and this response was boosted by the second dose. The highest neutralizing antibody titers were elicited by r80E protein formulated with the adjuvants AS05 or AS08 and protection against viremia was correlated with a higher neutralizing antibody titer at challenge. Recombinant sub-unit E proteins (80E) of the other DENV serotypes were produced using this same system. A tetravalent formulation of the recombinant 80E proteins was evaluated in mice and non-human primate experiments. In some instances, the NS1 protein of DENV-2 was included in the formulation to potentially enhance the immune response to the vaccine [50]. Macaques were immunized with the tetravalent formulation four times (day 0, 28, 67, and 102) and were challenged five months after the last dose. Due to the limited number of monkeys in each group, monkeys were only challenged with DENV-2 or DENV-4. Monkeys developed a robust neutralizing antibody response to all four DENV serotypes and were completely protected from DENV-2 challenge. The recombinant 80E subunit protein of DENV-1 adjuvanted with alum was recently evaluated in a Phase I clinical trial and preparations for a Phase I clinical trial of a tetravalent formulation is being planned [61]. Further development of this vaccine candidate was recently transferred from Hawaii Biotech to Merck and Co.

### 2.5. E protein Domain III Vaccine

The E protein of dengue is comprised of three domains, domain I, II, and III. Domain III contains the binding site for cellular receptors and antibodies directed against domain III have potent neutralizing activity [10,62,63]. Domain III contains serotype-specific and sub-specific epitopes that generate antibodies which neutralize the virus very well [64]. Vaccines containing only the E protein domain III epitopes are attractive because they would putatively be less likely to induce antibodies capable of causing enhanced viral entry upon subsequent wild-type dengue virus infection. Several candidate vaccines comprised of the domain III region of the dengue envelope protein have been evaluated in pre-clinical studies. One such vaccine incorporating the domain III of DENV-2 fused to the maltose-binding region of *E. coli* was evaluated in a prime boost protocol with a DNA vaccine as described above [29]. The domain III region of DENV-2 was fused with the meningococcal P64 protein (P64K) to generate a DENV-2 vaccine. Two fusion proteins were developed; one (PD3) inserted the domain III region into the lipoil-binding domain of P64K, the second (PD5) fused the domain III region to the C-terminus of P64K [51]. The entire P64K protein was required in order to obtain optimal folding, and therefore immunogenicity, of the DENV-2 protein [65]. Fifty micrograms of PD3 or PD5 (with CFA) were administered to rhesus macaques (3 animals per group) at 4 time-points; day 0, 30, 90 and 210. Animals were challenged with wild type DENV-2 45 days after the fourth dose. All animals had detectable neutralizing antibody after the fourth dose and 4/6 had detectable neutralizing antibody at challenge. All animals that received PD5 were completed protected from viremia following DENV-2, as were 2/3 animals in the PD3 group.

PD5 was further evaluated with either the outer membrane vesicle of N. meningitidis (OMV) or the capsular polysaccharide of serogroup A (CPS-A) as an adjuvant [54]. Non-human primates were immunized with one of the two formulations at day 0, 30, 90, and 150 and then challenged with wild type DENV-2 45 days after the last dose. Levels of neutralizing antibody were higher in the group of monkeys immunized PD5-CPS-A. The monkeys that received PD5-OMV had viremia titers following challenge that were similar to the control group; they were not protected. One animal that received PD5-CPS-A was completed protected against viremia following challenge and the second was partially protected [54].

A chimeric protein expressing the domain III E region and capsid protein of DENV-2 was mixed in a 3:1 protein:oligonucleotide ratio with 50-base-long DNA oligonucleotides in order to create aggregates of the antigens [52]. The capsid protein was included for proper folding of E. Both the aggregate and non-aggregate forms of the vaccine were evaluated in mice. Mice were immunized on day 0, 15, and 30 and were then challenged one month after the last dose with wild type DENV-2. Both forms of the vaccine induced similar titers of neutralizing antibody however, splenocytes isolated from mice immunized with the aggregate form had better secretion of IFN-γ than those isolated from mice immunized with the non-aggregate form of the vaccine. In addition, the aggregate form of the vaccine induced better survival from wild type challenge than did the non-aggregate form (70% *vs.* 20%). The authors speculated that the superior survival was due to cell mediated immunity induced by the aggregate form of the vaccine as both vaccines induced similar titers of neutralizing antibody [52]. The aggregate domain III-capsid vaccine, when administered 3 months after immunization with a live DENV-2 as part of a prime-boost approach, markedly boosted the neutralizing antibody response to DENV-2 in monkeys [53]. PBMCs collected from vaccinated monkeys 6 months after the booster dose secreted high levels of IFN-γ following stimulation with the domain III-capsid protein, indicative of a cell-mediated immune response. The authors propose that the domain III-capsid aggregate vaccine could be used in a prime-boost strategy with live attenuated vaccines resulting in an attractive immunization schedule as the vaccine was able to boost after only a three month interval.

## 3. Conclusions

Dengue has become the most important mosquito-borne virus in the world with more than 3 billion persons at risk annually for infection. As large epidemics of dengue continue to occur each year, the need for effective control measures becomes more urgent, and vaccines are considered a significant component. Although a live attenuated DENV vaccine candidate is in Phase III clinical evaluation, three doses of this vaccine must be given over a 12-month period of time to induce neutralizing antibodies to all four serotypes in flavivirus-naïve individuals. Several novel, second-generation DENV vaccine candidates have progressed through preclinical evaluation and have entered or will soon enter evaluation in humans. These vaccines are being developed to overcome some of the obstacles that live attenuated vaccines have encountered. Numerous DNA vaccines have been developed, and although the immune response induced by these vaccines was not as potent or as protective as live vaccines, these vaccines may be utilized as part of a heterologous prime-boost strategy. Antigens expressed by DNA vaccines have the potential advantages of both MHC class I and class II presentation, the ability to be administered to immunodeficient hosts, and the ability to induce a balanced immune response to all four DENV serotypes. When used as part of a prime-boost strategy, the dosing interval can be compressed such that multiple doses can be administered over a relatively short period of time.

Recombinant adenovirus vectored DENV vaccines are also being developed. These vaccines have many of the potential advantages of DNA vaccines, including the ability to be administered over a compressed dosing schedule (0 and 2 months). Additionally, they can generally induce more potent immune responses than naked DNA vaccines. Although there is a concern that pre-existing adenovirus antibodies in persons previously exposed to adenovirus could blunt the response to a rAd DENV vaccine, rhesus macaques that developed adenovirus antibodies after the first dose of a rAd DENV vaccine developed a good booster response to the DENV antigen when given a second dose of rAd DENV vaccine two months following the first dose [43]. rAd DENV vaccines have been evaluated as stand-alone vaccines as well as part of a prime-boost strategy in preclinical studies. Based on the promising results of these studies, evaluation of rAd DENV vaccines in human clinical trials is anticipated.

A very promising approach for the development of a potent tetravalent DENV vaccine is the use of an alphavirus replicon to express the protective antigen of DENV. A potential advantage of this vaccine over live attenuated DENV vaccines is its ability to induce neutralizing antibody against DENV when the vaccine is administered in the presence of maternal antibody. Alphavirus VRPs have also been evaluated as part of a prime-boost strategy to induce broadly neutralizing antibodies to all four DENV serotypes when given in a compressed administration schedule.

Many sub-unit protein vaccines have been evaluated in preclinical animal models and some are approaching or have been evaluated in clinical trials. Recombinant E protein domain III vaccines have been evaluated in non-human primate studies and have successfully induced protection against viremia following wild-type DENV challenge. These vaccines have potential in prime-boost protocols as they can be given as early as 3 months following a live vaccine. Finally, the sub-unit protein 80E vaccine was shown to be immunogenic and protective when given as a monovalent or tetravalent formulation in preclinical evaluation. This candidate is rapidly advancing; a Phase I clinical trial of a monovalent formulation has been completed and a Phase I clinical trial of a tetravalent formulation is being planned. As these promising next-generation DENV vaccines make their way into and through clinical trials, it is hoped that they will be able to overcome the hurdle of viral interference and induce potent neutralizing antibody responses in DENV-naïve individuals in a relatively short dosing interval.

## Figures and Tables

**Table 1.** Next-generation dengue vaccines in development.

**Vaccine**	**State of Development**	**Reference**
**Naked DNA vaccines—monovalent**	Preclinical Phase I clinical trial	[27–38]
**Naked DNA vaccines—tetravalent**	Preclinical	[39,40]
**DNA “shuffle” vaccines**	Preclinical	[41,42]
**Recombinant adenovirus vaccines**	Preclinical	[43–46]
**Alphavirus replicons**	Preclinical	[47,48]
**Sub-unit protein vaccine**	Phase I clinical trial	[49,50]
**E protein domain III**	Preclinical	[51–54]
